# Case Report: Severe osteoporosis misunderstood by bone metastasis after total gastrectomy and multiple metastasectomy

**DOI:** 10.3389/fonc.2023.1216705

**Published:** 2023-07-07

**Authors:** Seong Ho Hwang, Dong Jin Kim

**Affiliations:** ^1^ Department of Surgery, Daejeon St. Mary’s Hospital, College of Medicine, The Catholic University of Korea, Seoul, Republic of Korea; ^2^ Department of Surgery, Eunpyeong St. Mary’s Hospital, College of Medicine, The Catholic University of Korea, Seoul, Republic of Korea

**Keywords:** gastric cancer, osteoporosis, bone metastasis, nutritional screening, metastasectomy, local recurrence, total gastrectomy

## Abstract

After radical gastrectomy for gastric cancer, patients should be monitored from two perspectives. One is local recurrence or metastasis, and the other is nutritional and metabolic side effects. Herein, we report a case of severe osteoporosis that was misunderstood for bone metastasis due to increased bone scan and positron emission tomography–computed tomography uptake in the patient who underwent total gastrectomy and consecutive multivisceral metastasectomy. She was administered bisphosphonates, calcium carbonate, and cholecalciferol. After 3 months, a follow-up bone scan revealed decreased intensity of hot-uptake lesions, healed fracture lesions, and eventually improved bone pain. This study supports the need for careful nutritional screening as well as cancer surveillance after gastrectomy for gastric cancer and the need for screening guidelines for bone metabolic diseases.

## Introduction

Radical gastrectomy for advanced gastric cancer is the only treatment that is anticipated to cure the disease. Following gastrectomy with or without combined resection of other organs, patients must be monitored from two perspectives. One is local recurrence or metastasis, and the other is nutritional and metabolic side effects.

In gastric cancer, liver metastasis is the most common, followed by peritoneal and lung metastases. Conversely, bone metastasis is relatively rare (1). If bone metastasis is found, palliative chemotherapy or radiotherapy is usually administered; however, the prognosis is poor, and the quality of life is aggravated (2–4).

Nutritional and metabolic complications after gastrectomy are common and include weight loss (30%–84%), anemia (30%–60%), and bony disease (15%–30%) (5). After gastrectomy, food, bile, and pancreatic enzymes do not mix well in the intestines. Therefore, fat and fat-soluble vitamin D absorption is reduced, resulting in vitamin D deficiency. In addition, calcium absorption in the duodenum is also reduced, which eventually leads to osteoporosis (6, 7).

Long-term survivors of curative gastrectomy for stomach cancer often develop osteoporosis. The incidence of osteoporosis in patients who survive for >5 years after gastrectomy for gastric cancer is approximately 34% (8). However, while osteopenia or osteoporotic bone metabolic disease is relatively common, spontaneous microbony fractures due to severe osteoporosis are extremely rare. In that case, a bone scan or positron emission tomography–computed tomography (PET-CT) might detect active uptake lesions as bone metastasis. Although a bone scan is a highly sensitive test for bone diseases, it does not provide accurate information regarding its nature (9, 10).

In particular, if other conditions limit calcium absorption, the aggravation of osteoporosis can lead to spontaneous fracture (11), and positive findings are detected with bone scans or PET-CT. Herein, we report a case of severe osteoporosis that was misunderstood for bone metastasis due to increased bone scan and PET-CT uptake in a patient who underwent total gastrectomy and consecutive multivisceral metastasectomy.

## Case description

A 38-year-old woman underwent laparoscopic distal gastrectomy with Billroth II anastomosis for advanced gastric cancer in July 2006 (pT3N0M0, stage IIA, based on the American Joint Commission on Cancer Staging System 8th edition). The histological type was signet ring cell carcinoma, the depth of invasion was the subserosal layer, and the proximal resection margin was 2 cm.

In July 2007, the patient underwent completion total gastrectomy with distal pancreatectomy and splenectomy for remnant gastric cancer (pT4bN0M0, stage IIIA, based on the American Joint Commission on Cancer Staging System, 8th edition). The histological type was a tubular adenocarcinoma that is poorly differentiated and involved the pancreas. After surgery, the patient received six cycles of adjuvant chemotherapy with a combination of 5-fluorouracil and cisplatin.

During follow-up, dysphagia developed approximately 2 years later. Recurrence at the jejunojejunostomy site was found on CT and barium swallow series. In July 2009, after resection from the esophagojejunostomy to the jejunojejunostomy, including recurrent tumors, a Roux-en-Y esophagojejunostomy was newly created. The patient refused adjuvant chemotherapy, and only follow-up observation was performed.

However, 3 years later, a tumor was found in the left adrenal gland on follow-up CT, and fluorodeoxyglucose (FDG) uptake increased on PET-CT. The fourth surgery was performed in May 2012. Radical nephrectomy, adrenalectomy, Roux limb resection, and esophagojejunostomy were performed because the adrenal tumor invaded the left kidney, jejunal mesentery, and mesocolon. Histopathological examination confirmed metastatic signet ring cell carcinoma of the adrenal gland without lymph node metastasis. After surgery, the patient received eight cycles of S-1 adjuvant chemotherapy.

## Diagnostic assessment, details of the therapeutic intervention, follow-up, and outcomes

**Figure 1:**
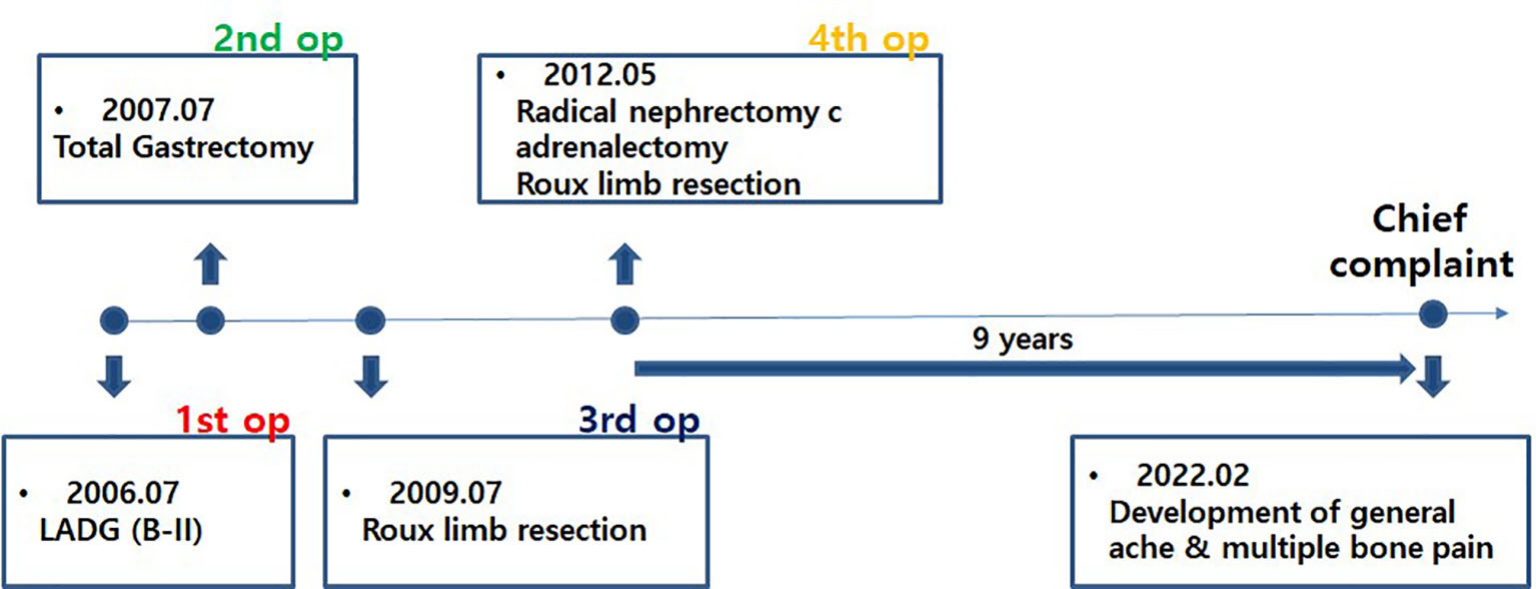
Timeline from the first surgery to the most recent onset of new symptoms. After undergoing a total of four surgeries over 6 years, the patient did well for 9 years without recurrence of metastasis and then visited our hospital again with new symptoms.

**Figure 2:**
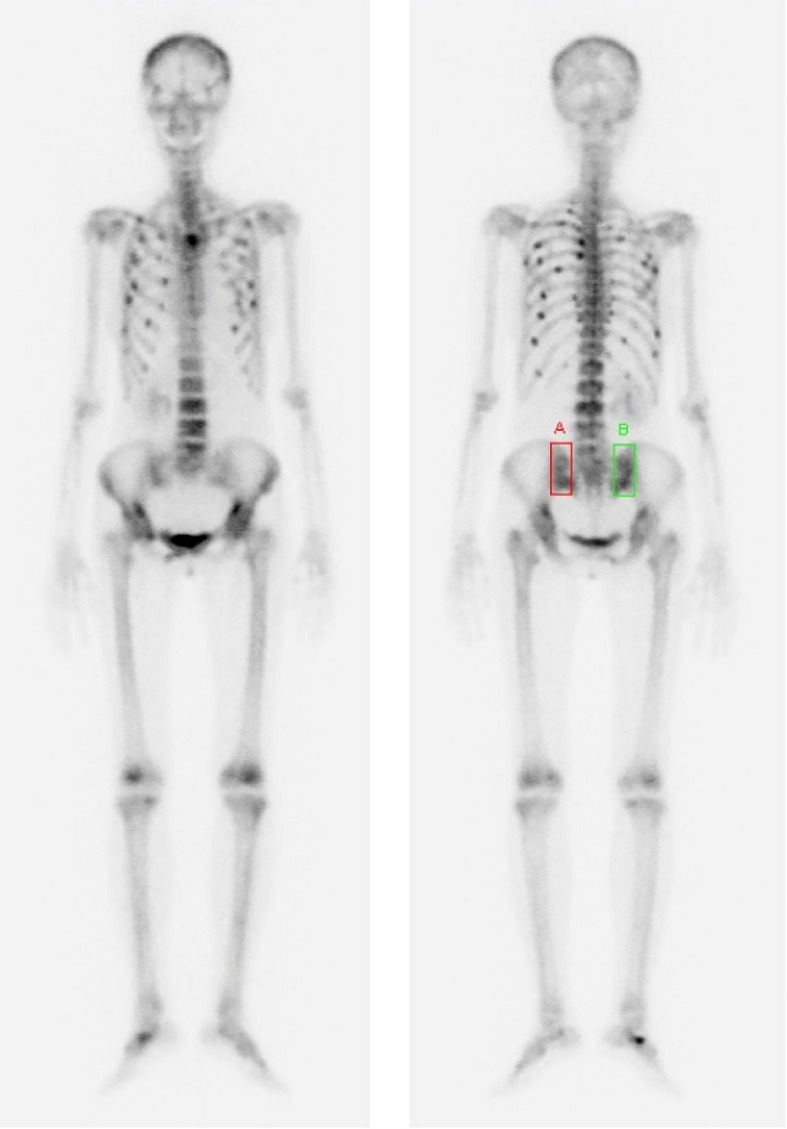
Bone scan images were performed to evaluate the cause of worsening general pain and multiple bone pain that occurred 9 years after the last surgery. These images showed multiple hot-uptake lesions suggesting bone metastases or multiple traumatic fractures in the spine, pelvis, and ribs.

**Figure 3:**
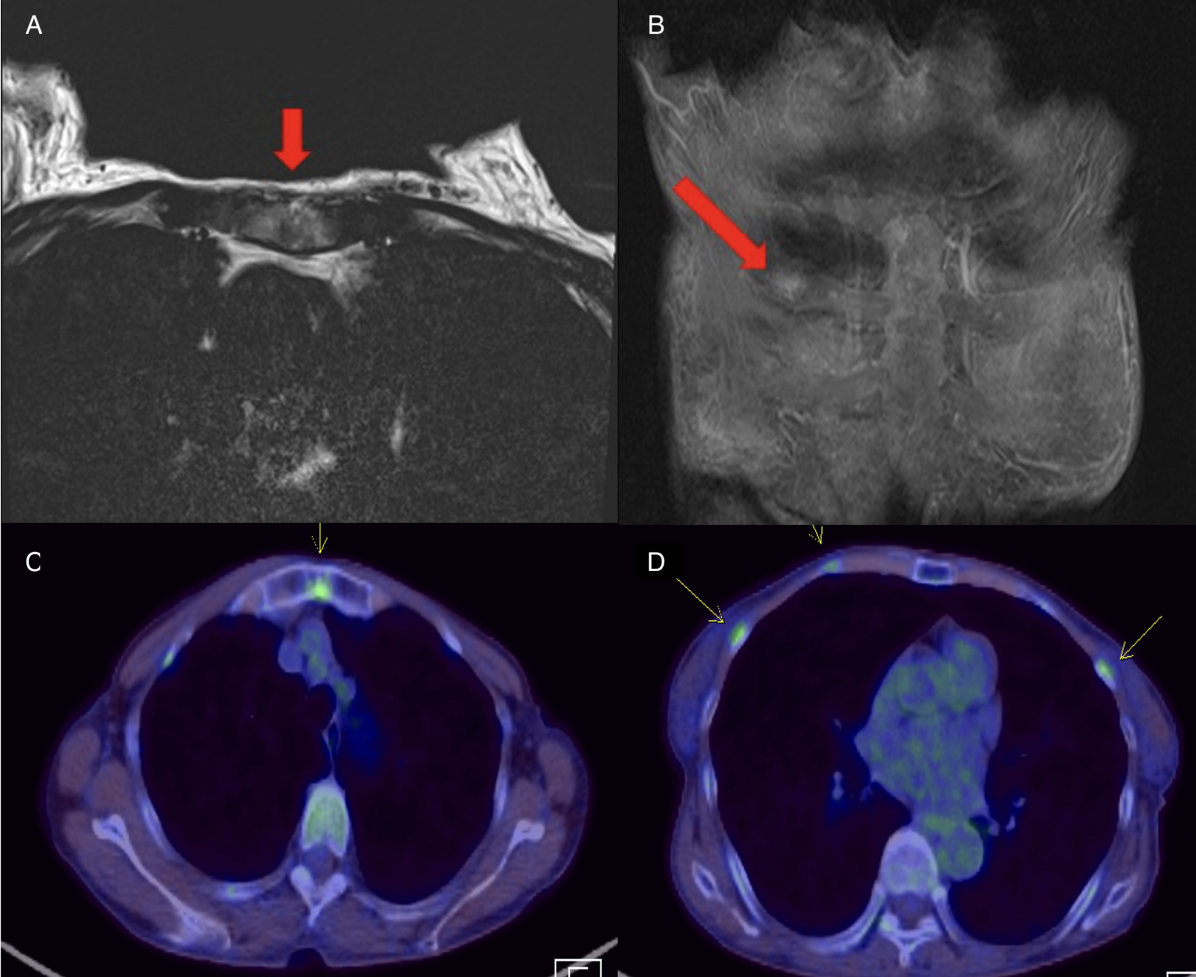
**(A, B)** Chest MRI scans. Red arrows indicate high-signal-intensity lesions at the sternum and right third rib. **(C, D)** PET-CT scans. Yellow arrows indicate multiple FDG uptakes in the sternum and ribs. High-signal-intensity lesions on MRI were consistent with lesions with FDG uptake on PET-CT. MRI, magnetic resonance imaging; PET-CT, positron emission tomography–computed tomography; FDG, fluorodeoxyglucose.

**Figure 4:**
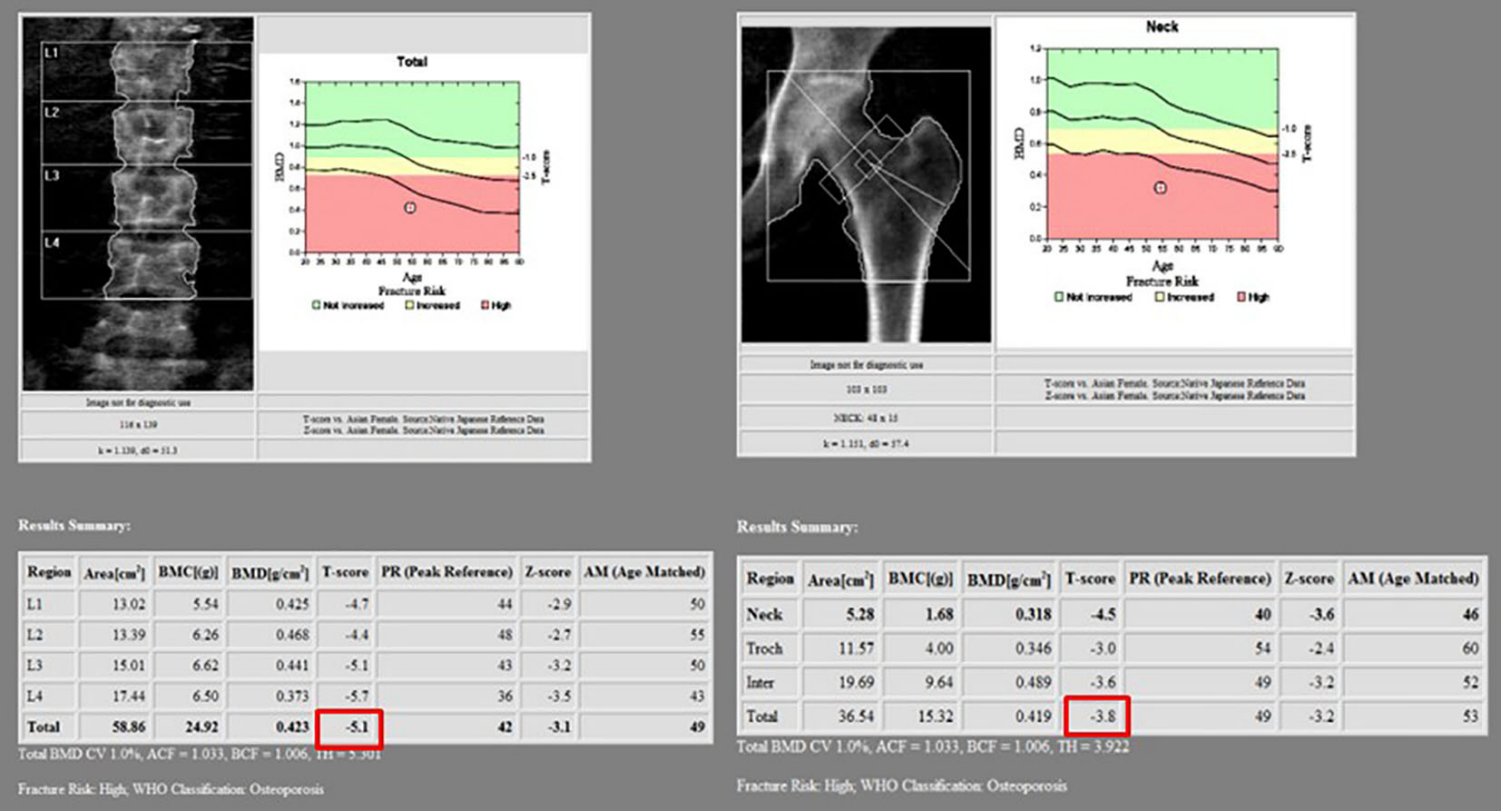
In the bone mineral density test, T-scores of the L spine and femur were less than −2.5, corresponding to osteoporosis. The total T-scores of L spine and femur were −5.1 and −3.8, respectively.

In February 2022, 9 years later, the patient visited our hospital again with worsening general aches and multiple bone pain (**Figure 1**). A bone scan showed multiple hot-uptake lesions on the rib and sternum, suggesting osteoporotic fractures or bone metastases (**Figure 2**). PET-CT confirmed FDG uptake similar to the bone scan (**Figure 3**). Additional sternal magnetic resonance imaging (MRI) revealed a focal enhancing lesion on the right third rib, suggesting bone metastasis (**Figure 4**). Therefore, rib segmental resection is required to differentiate between bone metastases and fractures.

However, the patient was afraid of an invasive procedure because of undergoing several difficult surgeries in the past and wanted to avoid it. Therefore, we decided to approach and manage her multiple bony fractures first, and invasive procedures or anti-tumor treatment were delayed until disease aggravation.

Further evaluation of bony metabolism was performed. As a result of laboratory tests, calcium was 8.0 mg/dl (normal range, 8.8–10.6 mg/dl); phosphorus, 2.2 mg/dl (normal range, 2.5–4.5 mg/dl); and total 25(OH) vitamin D, <5.00 ng/ml (deficiency, <20 ng/ml; sufficiency, 30–100 ng/ml). In the bone mineral density test, the T-score was −5.1 in the L spine and −3.8 in the femur neck, indicating that it corresponds to osteoporosis. Therefore, bisphosphonate, calcium carbonate, and cholecalciferol were administered.

After 3 months of treatment, laboratory test results improved to 9.0 mg/dl for calcium, 4.8 mg/dl for phosphorus, and 17.7 ng/ml for total 25(OH) vitamin D. In addition, compared with the previous bone scan findings, the follow-up bone scan showed a decreased intensity of hot-uptake lesions, and the fracture lesions healed. Eventually, bone pain improved (**Supplementary Figure 1**).

## Discussion

After gastrectomy, metabolic bone disease occurs due to vitamin D deficiency and decreased calcium absorption. However, as this metabolic bone disease progresses slowly, symptoms do not begin to appear until several years after gastrectomy. Therefore, osteoporosis is a common nutritional problem in patients with gastric cancer who survive long after gastrectomy. The patient in the case report was a long-term survivor of gastric cancer after gastrectomy; therefore, the risk of metabolic bone disease was high. However, as three previously recurred, the possibility of osteoporosis was missed because the concern for bone metastasis was greater.

The patient was a 54-year-old woman who underwent menopause 7 years ago and underwent total gastrectomy for gastric cancer. Therefore, she was at a higher risk for osteoporosis than the general population. In addition, multiple Roux-limb resections shortened the jejunum, resulting in reduced vitamin D absorption and impaired vitamin D metabolism due to nephrectomy. Generally, vitamin D is absorbed through the gastrointestinal (GI) tract and converted to vitamin D3 under ultraviolet light. Subsequently, vitamin D is converted to 25-hydroxycholecalciferol in the liver. Finally, in the kidneys, it is converted to its active form, 1.25-dihydrocholecalciferol. Vitamin D promotes calcium absorption at the intestinal level, increases bone mineralization, and maintains calcium homeostasis in the bones (12).

National cancer screening programs have recently been initiated, and most gastric cancers are diagnosed and treated at an early stage. In Korea, the 5-year relative survival rate for gastric cancer is >77% (13). Early detection and treatment of recurrence and metastasis are important during the survival period of patients with gastric cancer; however, long-term health problems during the extended survival period have also become important. Accordingly, interests have recently increased in approaching and appropriately managing health problems, improving patients’ quality of life, and reducing socioeconomic costs related to complications during the long-term survival period.

Long-term survivors of gastrectomy for gastric cancer can experience various health problems (14, 15). Osteoporosis can lead to complications such as fractures, chronic pain, reduced quality of life, and increased mortality (16, 17). Nutritional screening and management are required to prevent these complications in patients with gastric cancer. Currently, various guidelines are available for the diagnosis and treatment of gastric cancer. Among these are recommendations for bone metabolic disorders that can occur after gastrectomy for gastric cancer.

The European Society for Medical Oncology (ESMO) guidelines recommend follow-up tailored to the individual patient, disease stage, and dietary support, with attention to vitamin and mineral deficiencies (18). However, this study did not suggest a specific diet, vitamin, or mineral supplementation. The Korean practice guidelines for gastric cancer suggest that dual-energy X-ray absorptiometry can be used for the quantitative evaluation of bone mineral content and screening for osteoporosis. In general, oral calcium and vitamin D supplementation is recommended for populations at high risk of osteoporosis (19). However, this guideline also did not provide specific methods of oral calcium and vitamin D supplementation and pointed out that no universal guidelines are currently available for the prevention or management of metabolic bone disorders related to gastrectomy. Among the nutrition guidelines, the European Society for Clinical Nutrition and Metabolism (ESPEN) guidelines suggest general trace element and vitamin supplementation in patients with cancer patients (20). However, the guidelines were for nutritional support for patients with cancer in general, and patients who underwent gastrectomy for gastric cancer were not specifically mentioned. In particular, they were not guidelines for nutritional support for bone metabolism disorders.

Several studies have investigated severe osteoporosis after gastrectomy for gastric cancer. Most studies have investigated changes in bone metabolism by comparing bone mineral density with calcium, phosphorus, and parathyroid hormone levels (6). In addition, studies have been conducted to predict risk factors for osteoporosis. Recently, a nomogram has been developed to predict the risk of osteoporosis after gastrectomy for gastric cancer (21). However, we did not find any cases of severe osteoporosis that were misunderstood as recurrence due to a history of multiple surgeries for gastric cancer recurrence.

Previous studies have suggested that osteoporosis contributes to bone metastasis. In osteoporosis, increased inflammatory factors facilitate hematogenous metastases to the bone, and increased growth factors could enrich the local microenvironment, promoting the growth of the metastatic mass (22, 23). Thus, untreated osteoporosis may accelerate the progression of bone metastasis when it occurs (24). These studies are another reason that supports the importance of preventing and treating osteoporosis in long-term gastric cancer survivors who have undergone gastrectomy.

Patients who undergo gastrectomy for gastric cancer are at high risk of developing nutritional and metabolic complications. In particular, patients who undergo additional resections owing to recurrence should undergo nutritional screening and cancer surveillance. Therefore, nutritional and metabolic complications must be prevented. However, screening guidelines for osteoporosis in patients who underwent gastrectomy for gastric cancer have not yet been established. This study supports the need for screening guidelines for bone metabolic diseases in patients who have undergone gastrectomy for gastric cancer.

## Data availability statement

The original contributions presented in the study are included in the article/**Supplementary Material**. Further inquiries can be directed to the corresponding author.

## Ethics statement

The studies involving human participants were reviewed and approved by the Institutional Review Board of Eunpyeong St. Mary’s Hospital (IRB number: PC23ZIDI0007). Written informed consent for participation was not required for this study in accordance with national legislation and institutional requirements. Written informed consent was obtained from the participant/patient(s) for the publication of this case report.

## Author contributions

All authors contributed to the article and approved the submitted version.

## Supplementary material

Supplementary Figure 1 Bone scan images after 3 months of medication. Comparison of bone scans before and after medication showed a decrease in uptake of lesions, healing of fractures, and loss of bone metastases.
