# Supplementary figures and images for: Case Report: Severe osteoporosis misunderstood by bone metastasis after total gastrectomy and multiple metastasectomy
Source: Front Oncol. 2023 Jul 7;13:1216705. doi: 10.3389/fonc.2023.1216705 (PMC10361762; doi:10.3389/fonc.2023.1216705)

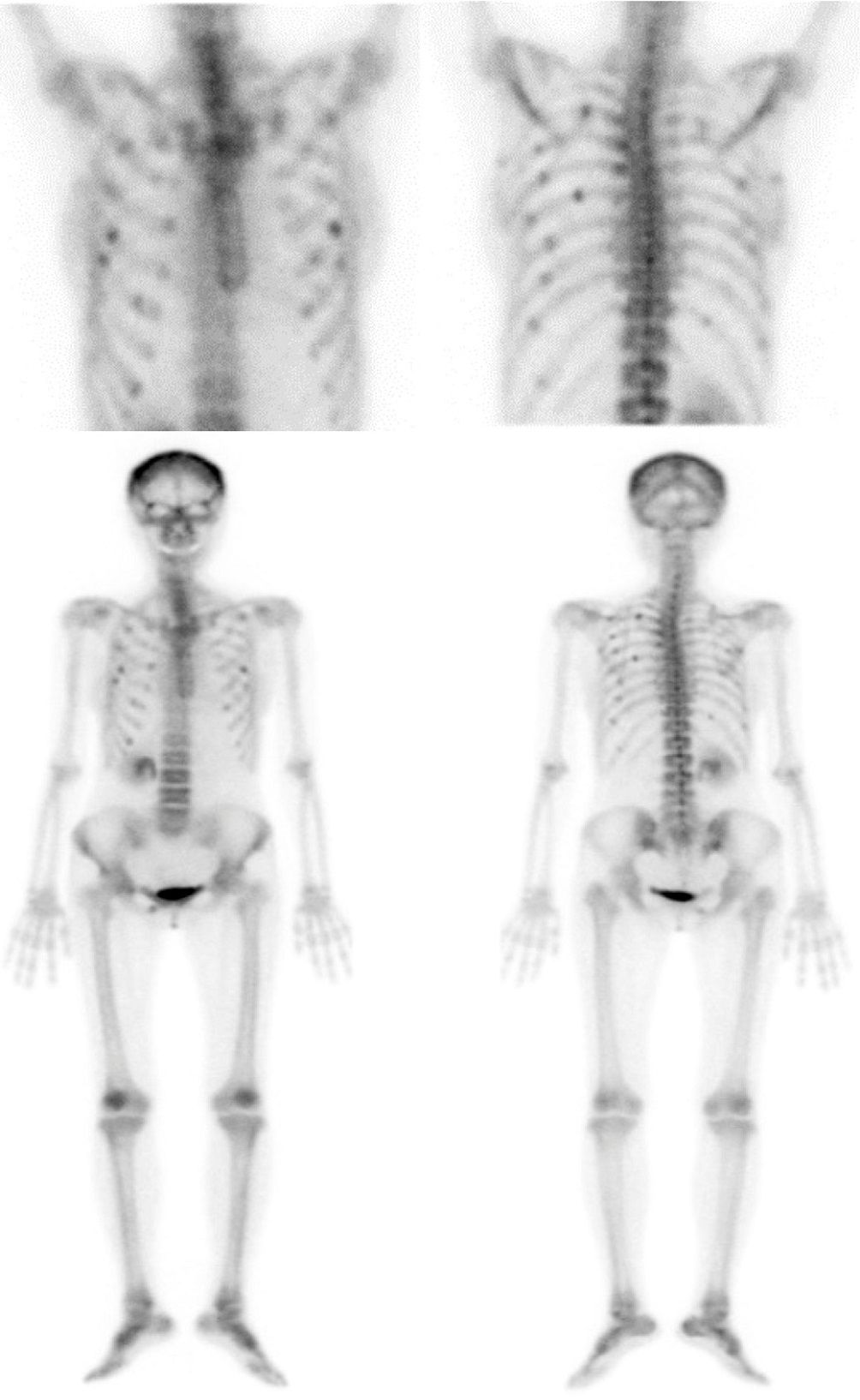

Supplement: Supplementary Figure 1 — Bone scan images after 3 months of medication. Comparison of bone scans before and after medication showed a decrease in uptake of lesions, healing of fractures, and loss of bone metastases. [file Image_1.jpeg]
